# Complementarity of Digital Health and Peer Support: “This Is What’s Coming”

**DOI:** 10.3389/fcdhc.2021.646963

**Published:** 2021-09-24

**Authors:** Patrick Y. Tang, Janet Duni, Malinda M. Peeples, Sarah D. Kowitt, Nivedita L. Bhushan, Rebeccah L. Sokol, Edwin B. Fisher

**Affiliations:** ^1^ Peers for Progress, Department of Health Behavior, Gillings School of Global Public Health, University of North Carolina at Chapel Hill, Chapel Hill, NC, United States; ^2^ Population Health Team, Vanguard Medical Group, Verona, NJ, United States; ^3^ Clinical Services and Research, WellDoc, Inc., Columbia, MD, United States

**Keywords:** diabetes, digital health, peer support, coach, self-management, primary care, app, telephone

## Abstract

**Purpose:**

This study examined integration of peer support and a Food and Drug Administration-cleared, diabetes management app (DMA) in diabetes self-management support as a scalable model for those with type 2 diabetes mellitus (T2DM).

**Methods:**

Two lay health Coaches delivered telephone-based self-management support to adults (*N* = 43) with T2DM recruited through a primary group practice. Those eligible were offered no-cost access to DMA for the entire 6-month study. Coaches introduced DMA and contacted individuals by phone and text with frequency dependent on participant needs/preferences. DMA supported monitoring of blood glucose, carbohydrate intake, and medication use, as well as messaging personalized to participants’ medication regimens. Clinical data were extracted from DMA, electronic medical records, and Coaches’ records. Structured interviews of 12 participants, 2 Coaches, and 5 project staff were analyzed using deductive pre-identified codes (regarding adoptability, patterns of use, value added, complementarity, and sustainability) utilizing standard procedures for qualitative analysis.

**Results:**

Of the 43 participants, 38 (88.4%) enrolled in DMA. In general, participants used *both* DMA and lay health coaches, averaging 144.14 DMA entries (structured, e.g., medications, and free form, e.g., “ate at a restaurant” and “stressed”) and 5.86 coach contacts over the 6-month intervention. Correlation between DMA entries and coach contacts (*r* = .613, *p* < 0.001) was consistent with complementarity as were participants’ and coaches’ observations that (a) DMA facilitated recognition of patterns and provided reminders and suggestions to achieve self-management plans, whereas (b) coaching provided motivation and addressed challenges that emerged. Mean hemoglobin A1c (A1c) declined from 9.93% to 8.86% (*p* < 0.001), with no pattern of coaching or DMA use significantly related to reductions. Staff identified resources to coordinate coach/DMA interventions as a major sustainability challenge.

**Conclusions:**

DMA and peer support for diabetes management are compatible and complementary. Additional practice integration research is needed for adoption and scale-up.

## Introduction

Integration is important across many dimensions of diabetes care, such as individual self-management *behaviors* according to the ADCES7 Self-Care Behaviors^™^ (1) as well as across varied *sources* of care (2). This project developed and tested the integration of two sources of support: lay health coaching, idiomatically “soft touch,” and digital health support—“high tech”—for individuals with type 2 diabetes mellitus (T2DM).

Systematic reviews have documented the benefits of peer support for people with diabetes (primarily T2DM) (3–8), as well as many other health concerns (9, 10). Given effectiveness, the challenge for peer support in diabetes care is to develop models that are feasible, scalable, and cost-effective in real-world settings. At the same time, digital health platforms such as smartphone apps have shown effectiveness in diabetes prevention (11–13) and management (14–17), including a review of reviews that found average reductions of 0.5 points in glycated hemoglobin (A1c), mostly among those with T2DM (18). Digital health solutions support individuals in their daily self-management activities and provide valuable patient-generated data to providers to enhance shared decision-making regarding treatment decisions (19). The convenience and low cost of digital health appeal to people that might otherwise face financial, logistical, or communication barriers when participating in face-to-face interventions. The challenges for digital health include engaging and retaining users, and addressing complex needs (12, 20–22).

Evidence from these two areas of study point to the unrealized potential of well-integrated peer support and digital health. A systematic review of web-based strategies in diabetes self-management (20) noted that interventions incorporating peer support were more likely to be effective than those that did not. Furthermore, a meta-analysis found that diabetes mobile phone apps reduced A1c among individuals with type 2 diabetes and the effect size was associated with the amount of feedback from healthcare professionals (23), suggesting that apps can be more effective when there is someone to provide live support. Peer support can provide grounding for digital apps, promoting uptake and continued use by incorporating those tools into routine contacts. Reciprocally, digital health may provide data to prompt and guide the work of peer supporters, while also reducing the burden of routine tasks such as monitoring key behaviors and indicators like blood glucose. Indeed, a 2020 review of reviews and gap analysis examining in-person and technology mediated peer support (8) identified as a gap exploration of “technology-mediated peer support, beyond voice-based telephones … [and including] … technology-mediated peer support modalities (i.e., video conferencing, SMS text message, social media).”

This study focused on a diabetes management app (DMA) for T2DM developed by WellDoc, Inc., a collaborator in the present project. The DMA had been cleared by the Food and Drug Administration. Using individuals’ own data (structured and free form, e.g., medications, blood glucose readings, carbohydrates, exercise, or “ate at a restaurant,” “stressed,” and “felt sick”), the DMA responds with adaptive messaging that is personalized to individuals’ medication regimens, and aligned with standards of care of the American Diabetes Association and the Association of Diabetes Care & Education Specialists (ADCES, formerly the American Association of Diabetes Educators) (24, 25). It also offers messaging and sharing clinical reports with individuals’ providers to enhance patient-provider communications and shared decision-making. Randomized controlled trials of the DMA software demonstrated significant reductions in A1c of 1.9 and 2.0 points in intervention groups (26, 27). At the time of the present project, the DMA was available by prescription but provided free to participants in the study. It is now also available without prescription.

The present project developed and assessed the offer of peer support and the DMA for self-management education and support for adults with T2DM in a primary care group practice setting. Drawing from the expanding literatures in implementation and dissemination research (28–30), key points of evaluation included (a) patterns of use, including how much participants would utilize each of peer support and DMA, (b) clinical changes and participants’ as well as staff observations regarding the value added by the combination, (c) participant and staff observations as well as patterns of use regarding the complementarity of peer support and DMA, and (d) staff comments regarding the sustainability of the combination within their clinical setting.

Complementarity has many meanings including in common language in which, say, one item of clothing is said to complement, that is, go well with, another. In behavioral economics (31), complementarity refers to a positive association between consumption of two commodities, e.g., bread and butter, in response to price changes for either. As price changes alter the consumption of one, the consumption of the other will change in the same direction. That is, reducing the price of bread leads to increased consumption of not only bread but also butter. Both of these share the sense that things “go together.” It is in that sense that we consider complementarity here, that is that use of the DMA and engagement with the Coaches might tend to go together, in contrast to one replacing the other, “substitutability” in behavioral economics terms.

## Methods

### Research Setting

In 2015, Peers for Progress in the Gillings School of Global Public Health at the University of North Carolina-Chapel Hill (UNC-CH), WellDoc, Inc., and Vanguard Medical Group in New Jersey (Vanguard) joined to pursue this project. Vanguard’s eight primary care sites are recognized patient-centered medical homes with care coordination processes to manage high-risk individuals, electronic medical records (EMRs), disease registries, quality metrics tracking, patient portal, and online appointment scheduling. Vanguard’s clinical staff were involved throughout the project to ensure feasibility. The study was approved by the Institutional Review Board at UNC-CH. Vanguard, WellDoc, and UNC-CH entered into a data use agreement to facilitate data sharing and analysis.

### Formative Evaluation and Intervention Development

UNC-CH researchers conducted a formative evaluation in June–July 2015, following standard health education methods (32), to determine the feasibility of combining peer support and digital health. Telephone interviews were conducted with 4 care coordinators, 1 physician’s assistant, and 4 patients with T2DM at Vanguard (33). Vanguard patients each received a $30 gift card for their participation in the interviews. They ranged in age from 31 to 73.

As previously described (33), participants noted potential value in a diabetes self-management intervention that employed some combination of DMA and live peer support. Contrary to possible expectations that individuals might object to having their choices and activities guided by an automated or artificial system, no one expressed such objections to using the DMA. Clinical staff saw value in the proposed intervention but expressed concerns about the potential of lay health coaches (Coaches) promoting inaccurate medical information and the lack of coordination with clinical care. The concerns of clinical staff were addressed by employing a certified diabetes educator to assist in training the Coaches, conducting weekly supervisory meetings with the entire study team, and coordinating the activity of Coaches through the nurse care coordinator (JD).

Following the formative evaluation, the study team developed the intervention according to the following principles: (1) the DMA would assist individuals with daily self-management and provide regular encouragement and praise, (2) the Coaches would work with participants to provide social support and assist with problem-solving and overcoming challenges, (3) the Coaches would also encourage participants to utilize a variety of DMA features, (4) participants were free to determine the extent to which they utilized health coaching and the DMA, and (5) data entered by participants into the DMA would prompt timely follow-up from the coaches.

### Recruitment and Training of Lay Health Coaches

Two lay health coaches (Coaches) were recruited through the department of nutrition at a local university. Both had bachelor’s degrees but had not been previously employed in healthcare. The Coaches were employed through UNC-CH with paid, part-time positions (15–20 h per week).

The initial two-day training (18h) was held onsite at Vanguard, covering basic diabetes education, self-management support, effective peer support, communication skills, the DMA, research ethics, coaching protocol, and study documentation. The training was led by a certified diabetes educator and nurse coordinator from Vanguard, a DMA trainer from WellDoc, and trainers from Peers for Progress. The Coaches each received a smartphone on a prepaid plan with a local number to carry out the intervention.

### Participant Recruitment and DMA Onboarding

Vanguard identified 203 potential participants from four of its eight primary care sites according to the following criteria: T2DM, 30–75 years of age, and at least one A1c value ≥ 7.5% in the previous 12 months. Although identified by A1c ≥ 7.5%, individuals were contacted in descending order of A1c values in order to recruit a sample of greatest clinical need. During recruitment, Coaches confirmed that participants were fluent in English and had regular access to a web-enabled device (smartphone or computer).

Each potential participant received a mailing containing a signed invitation letter from their primary care provider, HIPAA and consent forms, and a stamped return envelope. Coaches commenced telephone recruitment 2 weeks after the mailings were sent out unless individuals had opted out. Coaches attempted a total of seven contacts, including voicemails and a personalized follow-up letter, before designating individuals unable to be reached. Upon successful contact, Coaches introduced the program, obtained verbal HIPAA authorization and consent, and conducted a health assessment. Participants were asked to return signed HIPAA and consent forms using the stamped return envelopes. Following successful recruitment, Coaches set up a second call to assist with DMA installation and registration with DMA customer care for further technical support.

### Coach Intervention

Beginning with the third call, Coaches began to build rapport with the participants and initiated substantive discussions around self-management. They inquired about the participant’s general health status, answered questions about using the DMA, and assisted participants with entering data into the DMA. Coaches probed emotional status using items from the Diabetes Distress Scale (34, 35) and reviewed individuals’ diabetes self-management, such as their familiarity with A1c and fasting blood glucose measures, self-monitoring blood glucose, medication adherence, diet and exercise, and adherence to recommended clinic visits and examinations. If neither the participant nor DMA data suggested a self-management target, Coaches reviewed the ADCES7 Self-Care Behaviors™ with participants to help set goals. Coaches encouraged the participant to attend diabetes self-management education if they had not already done so, and checked that the participant had a glucometer, testing strips, and prescribed medications.

Frequency of ongoing coaching calls ranged from biweekly to monthly according to participants’ needs and preferences. Each call began with follow-up on matters raised in the previous call as Coaches tailored discussions to individual participants. A protocol guided notification of the nurse coordinator in response to urgent medical issues or emotional distress. The intervention lasted up to 6 months, with individuals participating for 5–6 months depending on latency of enrollment.

The Coaches conducted their telephone calls from the care coordination office at Vanguard. This colocation facilitated back-up and coordination with the clinical team. Additionally, Vanguard, WellDoc, and UNC-CH staff held weekly calls with Coaches to address emergent issues, hot cases, and questions around protocols. This process led to improvements such as streamlined DMA onboarding, better incorporation of diabetes care standards, development of protocols for addressing urgent needs and strategies for motivating participants after the New Year, and guidance on generating discussions around participants’ own DMA data.

### Fidelity of Implementation

Fidelity of implementation was monitored and encouraged in several ways. As noted above, staff (JD, EF, MP, and PT) met weekly with the Coaches to review progress and discuss any adjustments to the protocol as well as emergent clinical concerns. Coaches followed detailed protocols and scripts for initial contact of potential participants including, e.g., number and timing of recruitment calls. As noted in *Results*, Coaches succeeded in onboarding to the DMA 38 of the 43 participants for whom this was intended (88.4%). Per protocol, however, contacts with the Coaches and DMA entries were left free to vary in order to assess participants’ utilization of these. Project staff monitored coaches’ timely submission of contact notes.

### Evaluation

Evaluation drew from established approaches to implementation and dissemination research including Glasgow’s RE-AIM model, Proctor’s implementation model, and the PRISM model (28–30). It also followed a triangulation design (36) that combined several investigators and interviewers (investigator triangulation) as well as both qualitative and quantitative data (methodological triangulation) (37) to address research questions comprehensively and to validate findings generated by each approach.

#### Qualitative Data Collection and Analysis

After the conclusion of the intervention, semi-structured interviews were conducted with 12 participants, 2 Coaches, and 5 project staff from UNC-CH, Vanguard, and WellDoc. Twelve participants, representing different patterns of participation in the program (both coaching and DMA, DMA with little coaching, and coaching with little DMA use), were recruited to participate in these interviews through their Coaches. Vanguard patients each received a $30 gift card for participating in the interviews.

UNC-CH graduate research assistants conducted the interviews by phone or in-person. Interviews probed strengths and challenges of the program, and solicited recommendations for dissemination, scale-up, and tailoring (28, 38). Interviews lasted 20–70 min, and were audiotaped, transcribed, and imported into Atlas.ti for analysis. Interviews were coded by three graduate research assistants according to deductive pre-identified codes and inductive emergent themes utilizing standard procedures for qualitative analysis (39).

#### Quantitative Data and Analysis

Baseline data included age, whether prescribed insulin, most recent A1c, blood pressure, and lipid values from EMR up to 10 months prior to initiation of the study. End-of-treatment values were the last available, up to 1 month following intervention termination. Data from DMA included blood glucose readings, carbohydrate counts, medication adherence, hypoglycemic events, and free text notes/diaries. Coaches’ records included dates of contacts and topics discussed.

Statistical methods used to describe the sample, patterns of use, and value added included descriptive statistics, and *t*-tests to assess differences between those who participated and those who were eligible but did not participate. Other differences are detailed in the *Results*, such as participation by sex. Principal components analysis examined the factorial structure among the number of different types of entries to the DMA. Analyses also included correlations among participation variables and between those variables and clinical indicators, and within-group *t*-tests of changes in clinical indicators.

## Results

Following methodological triangulation’s (36) inclusion of both qualitative and quantitative data (37), the quantitative and qualitative data are integrated in answering each of the research questions. **Table 1** includes illustrative quotations from the interviews, organized according to the key sections of the *Results*: (a) patterns of use, (b) the value added by the coach and DMA support, (c) the complementarity of coach support and DMA, and (d) sustainability within the clinical setting. Each section of the *Results* draws also from the interview results, including illustrative quotations from **Table 1**, indicated as from participants—“PQ”; Coaches—“CQ”; or staff—“SQ” and numbered consecutively.

**Table 1:** Quotations illustrating patterns of use and adoptability, value added, complementarity, and sustainability challenges from participants, coaches, and staff.

Category	Quotation
Patterns of Use—Adoptability	“I like that it was personal. I liked that it was conversational. Yes, there was some technology involved. I liked that there was someone who could give me correct, intelligent answers, rather than opinion.” (PQ1) “It should be a program that don’t end. It should continue. It should be the oath for the people, well I don’t know if everybody likes it, I know I like it. It should be there for people that want to do it.” (PQ2)
Value Added (support from Coach and BlueStar, behavior change)	“It was easy. Like I said, for me it was easy talking to [Coach]. It was easy phone conversations, communication. It wasn’t pushy but it was persistent. I felt comfortable with that. It was not like you’re going in and somebody’s pushing you to do this or pushing you to do that. To me, I guess, he found a level for me to communicate with and that’s why it was a comfortable thing” (PQ3) “He talked to me about so many things. One of the things I tell you that I’m going to be missing his phone call. He was really into it. The advice he gave me I took them and I feel good and the depression started going away once I started doing like he wanted.” (PQ4) “I never really have anybody follow up on me. I was always disappointed with my diabetes doctor because he was very lackadaisical and I changed in this year … With the coaching, Gene called me every once in a while and he reminded me that everyday life you’ve got to keep your eyes open to do the right thing. He was helpful that way.” (PQ5) “Well, before this program I was not doing exercise. That’s one for me. Second of all, I was not testing my sugar like I test them now. That’s another thing. Third, I was smoking and I stopped it where it would make me feel better. I stopped drinking caffeine and now I drink less. I drink more water than anything. Those kind of things, that changed a lot because before me getting into this program I was doing a lot of the other things. Like the smoking, not exercising, the caffeine and all that and now all that is gone.” (PQ6) “I have another patient who she started using a Fitbit during the project. She was able to sync the Fitbit with BlueStar, and really just super jazzed about the program, and being able to track everything that she does. She also prior to the program was not really checking her blood glucose that much, she just really is uncomfortable with the finger prick, so she does check it now, it’s not as often as maybe her provider would like. It’s maybe one or two times a week, but that’s a start, and that’s more than she was before it, so yeah, those specific patient’s really made some drastic improvements I think.” (CQ1)
Complementarity	“BlueStar kinda guides you and the coach is a good motivator.” (PQ7) “My conversations with the health coach were tied into BlueStar, but also tied into general wellness. I didn’t use BlueStar for general wellness, but I used my health coach for that.” (PQ8) “The BlueStar it’s okay because there you put numbers and whatever you are day-to-day. The coaching is different. The coaching is a person that is speaking to you. It’s someone that you’re listening to. It’s someone that is giving you advice. Like I said, other than reading. Let’s say like I said, ‘Now, I stopped smoking.’ I cannot put that in the BlueStar. It would tell me you could do this, this, this and that. Instead of the cigarette now you could do this and it would be about the same thing.” (PQ9) “I would characterize the role of BlueStar for my patients as a day-to-day tracker, so something that’s taking place of a paper log book for them, and that’s what they are primarily using to log their blood glucose, and to check off that they took their medications, and in some instances carb count, or calorie count. BlueStar would be more of the day-to-day, and the role of coaching would be to just reiterate how those day-to-day aspects within BlueStar are going, and discuss things that maybe aren’t so day-to-day, like checking in on if they need blood work, or a primary care provider visit, or checking in on their diet, or their exercise routine, which isn’t something that you can really grasp, and get support on through BlueStar I think.” (CQ2) “The only negative thing [about the intervention] was the computer access … I think that was a little cumbersome. If it went to phone, it might have been a little bit easier but I just couldn’t wrap my head around it and it was just kind of complicated. The phone calls and the support, the coaching, and that stuff was very good.” (PQ10)
Sustainability, Including Challenges to Sustainability	“I think scaling this up, we would need to add an entirely different staff member to the Vanguard offices, to liaison if we were going to use this same type of setup we have now. I’m not sure that that’s something that Vanguard would do.” (SQ1) Dissemination would need “More care coordinators … That’s always part of the difficulty is that a lot of the clinical people in the facility tend to be overworked and very busy.” (SQ2) “BlueStar has a built in model that has that sustainable. The issue is, does this combination program make it more affordable for the clinics to hire the coach? If you’re comparing it to just a coaching program by itself, without BlueStar and they can do 30 patients versus with BlueStar and they can handle 50 patients. I think that’s a great argument for being more sustainable than a traditional coaching program.” (SQ3) “[I]f the coaches could have had access to the EHR, I think they would have been able to mine more interesting data … I think it would have taken some of the heavy lift off the care coordinators and our medical assistant admin support people to go in and get the latest lab work, and print it for the coaches.” (SQ4) “I don’t look at it as it being bad about the program it’s just the challenges, the biggest challenges for me were some patients were very hard to reach. As a coach, in the beginning, that was harder because I want to try to help them and then they stayed, they elected to enroll so I’m assuming at that point that they, they were kind of like at least past the first stage of change or kind of thinking about changes. I guess the biggest challenge was, for me, and of the program as a whole, was trying to move people who were not even thinking about, not really thinking about changing from that stage to thinking about changing, that was the hardest thing for me.” (CQ3)

Quotations cited in the text are numbered sequentially and denoted as Participant Quotations—“PQ,” Coach Quotations—“CQ,” or Staff Quotations—“SQ.”

### Recruitment and Sample Characteristics

Of the 203 potential participants identified from EMR data, 140 were contacted for participation, of whom 46 were found to be ineligible. Of the 94 eligible, 47 declined and 47 (50%) consented to participate. This is a common rate of acceptance. For example, recruitment was 51.8% (240 of 463) in a pragmatic trial of the same DMA as used here that was also restricted to those with A1c levels at or above 8% and that included in-person identification by clinicians during regular clinic visits (40).

It should also be noted that individuals were approached for participation starting with those with the highest A1c values. Relative to the baseline A1c of 8.96 in the aforementioned study (40), this resulted in mean baseline A1c of 9.93% in each of the current groups that participated and that declined participation.

The study objective was to examine use of and benefits from the *availability* of both live coaching and DMA. Availability was operationalized as minimal exposure to each, leaving unconstrained variation in subsequent use. Minimal exposure was defined as two coach contacts in the second of which DMA onboarding was planned. Of the 47 who consented to participate, 43 (91.5%) met this criterion of two coach contacts, among whom 38 (88.4%) enrolled in DMA.

**Table 2** compares pre-intervention values for these 43 participants with those of 114 from among the 203 identified from EMR as eligible but excluding the 46 confirmed to be ineligible during initial recruitment. Thus, the 114 to whom the 43 participants are compared includes four who agreed to participate but did not meet the criterion of two coach contacts, 47 who were reached but declined, and 63 who were not reached, some of whom might have been found ineligible had they been reached. There were no appreciable differences between the groups (*p*s for *t*-tests ranged from 0.365–0.993).

**Table 2:** Comparisons of participants and nonparticipants: percentages or means (standard deviations) on key variables pre-intervention.

Variable	Participants	Nonparticipants	Total
Age	57.12 (9.23)	55.65 (10.57)	56.05 (10.22)
	*N* = 43	*N* = 114	*N* = 157
% Female	48.8%	39.5%	42.0%
	*N* = 43	*N* = 114	*N* = 157
% on Insulin	37.8%	30.2%	35.7%
	*N* = 43	*N* = 111	*N* = 154
Pre-A1c	9.93% (1.28)	9.93% (1.50)	9.93% (1.44)
	*N* = 42	*N* = 112	*N* = 154
Pre-BMI	33.24 (6.74)	34.00 (6.55)	33.78 (6.59)
	*N* = 42	*N* = 103	*N* = 145
Pre-Systolic Blood Pressure	126.56 mm Hg (12.46)	127.34 mm Hg (11.85)	127.12 mm Hg (11.99)
	*N* = 43	*N* = 109	*N* = 152
Pre-Diastolic Blood Pressure	77.61 mm Hg (9.33)	78.25 mm Hg (8.68)	78.07 mm Hg (8.84)
	*N* = 43	*N* = 109	*N* = 152
Pre-Low-Density Lipoprotein	100.67 (27.34)	98.53 (34.28)	99.15 (32.35)
	*N* = 37	*N* = 92	*N* = 129
Pre-High-Density Lipoprotein	46.96 (11.98)	46.38 (14.65)	46.55 (13.87)
	*N* = 41	*N* = 98	*N* = 139

Participants’ healthcare was covered by commercial insurance or Medicare. From zip codes of participants, estimated per-capita income was $42,126. In the third quarter of 2015 when participants were recruited, the per-capita income in New Jersey was $61,136 (41), 45.1% or almost $20,000 higher than that of participants.

### Patterns of Use

Among the 43 participants, coach contacts and DMA entries are described in **Table 3**. Coach contacts ranged from 1 to 16 with an average of 5.86. Of the 38 who enrolled in DMA, 29% utilized the DMA with smartphone only, 29% with computer only, and 42% with both. Six participants (16%) entered at least one hypoglycemic value (BG < 70 mg/dl) and 8 (21.1%) entered at least one hyperglycemic value (>300 mg/dl), both of which triggered real-time feedback within the DMA. Fourteen participants (36.8%) transmitted at least one “SMART Visit Report” (integrated DMA data including glucose control and self-management behaviors) to their Vanguard PCP for review at their office visit.

**Table 3:** Characteristics of patterns of coach contacts and DMA use among 43 participants.

Characteristics	Minimum	Maximum	Mean	Std. Deviation
Coach Contacts	1.00	16.00	5.86	3.02
Number of blood glucose entries	0	952	70.23	161.50
Number of carbohydrate entries	0	150	15.70	35.33
Number of medication entries	0	360	44.28	78.67
Number of note entries	0	154	14.93	37.84
Total BS Entries	.00	1,443.00	144.14	270.11

Ninety-three percent of coach contacts were initiated by the Coaches. Their contact notes provided data on the topics of contacts, including the percentage of contacts that included discussion of each of the ADCES7 Self-Care Behaviors™ (1). These were healthy eating—65.6% of contacts, physical activity—58.2% of contacts, glucose monitoring—67.7%, medication adherence—69.5%, reducing risks—62.8%, problem solving—65.3%, and healthy coping—63.5% of contacts. Turning to types of coaching support, the percentages of calls in which each of the following types of support were provided were as follows: emotional support—64.9%, encouragement or motivational support—82.1%, problem solving—42.5%, new goal(s) set—31.2%, and review of goal progress—46.7%. Support for PCP visits was included in 24.6% of contacts and miscellaneous topics (economic, legal, social, and health services) in 5.3%.

**Table 3** also provides the ranges and means of blood glucose, carbohydrate, and medication entries and numbers of notes into DMA. Principal components analysis indicated that they were best characterized as a single factor (Eigen value = 2.792, loadings ranging from .707 to .926). Consequently, these four types of entries were summed to create “total entries” that ranged from 0 to 1,443 with a mean of 144.140 (SD = 270.114).

There were no significant relationships between participant characteristics (age, baseline SBP, DBP, and A1c) and number of either DMA entries or coach contacts (*p*s range from 0.372 to 0.993). The 13 participants with prescriptions for insulin averaged twice as many total entries into DMA compared to the 30 without insulin prescriptions, 225.23 *vs*. 110.43, but this was not significant (*p* = 0.204) because of large variability (SD = 270.11). Similarly, those with insulin prescriptions averaged 6.846 coach contacts versus 5.433 for those without insulin prescriptions, but this also was not significant (*p* = 0.161). There were no significant differences by sex in DMA entries (*p* = 0.789) or coach contacts (*p* = 0.624).

Participants, Coaches, and study staff spoke positively about the program. Participants mentioned the flexibility and user-friendliness of DMA and that there was live coaching from “someone who could give me correct, intelligent answers” (PQ1) and of the value of ongoing support, “It should be a program that don’t end” (PQ2).

### Value Added

Among the 43 participants, pre- and post-A1c values were missing for one participant and post values were missing for an additional four. For these four, post values were conservatively imputed as equal to pre values, resulting in an analytic sample of 42. A1c declined from 9.93% (SD = 1.28), reflecting the selection of participants on the basis of high A1c scores, to 8.86% (SD = 1.84) after the end of the coach intervention (*t* = 4.09, df = 41, *p* < 0.001). Among the 37 who both enrolled in DMA and had at least two coach contacts, the change was similar (9.99, SD = 1.32, to 8.80%, SD = 1.93, *t* = 4.21, df = 36, *p* < 0.001). Using categories from the National Committee for Quality Assurance (42), the percentage with A1c below 9% increased from 27.9 to 55.8% and that below 8% increased from 0 to 33.3%.

We divided the 43 participants at/above or below the median for coach contacts (median = 6) and total DMA entries (median = 21). **Table 4** shows that for each combination of high or low use of Coach and DMA, A1c values declined a minimum of 0.49 points (9.69% to 9.20%). Although not significant because of small sample sizes, the smallest reduction, 0.49 points, was among those relatively low on *both* DMA entries and coach contacts.

**Table 4:** A1c means and mean changes from pre- to post-assessment disaggregated by level of BlueStar entries (low/high) and numbers of coach contacts (low/high).

BlueStar Entries and Coach Contacts	Pre-A1c (Std Dev)	Post-A1c (Std Dev)	A1c Change (Std Error)
Low BlueStar			
Low Contacts	9.69%	9.20%	0.49
*N* = 13	(1.367)	(1.974)	(0.504)
Low BlueStar			
High Contacts	10.38%	9.25%	1.13
*N* = 8	(1.166)	(2.063)	(0.597)
High BlueStar			
Low Contacts	10.78%	9.23%	1.55
*N* = 6	(1.105)	(2.279)	(0.614)
High BlueStar			
High Contacts	9.56%	8.21%	1.35
*N* = 15	(1.213)	(1.393)	(0.436)

Participants cited the support, guidance, knowledge, and motivation received from their Coach and overall motivation that the program provided. When describing support received from the Coaches, participants described it as “wasn’t pushy but it was persistent” (PQ3), “someone who was there for me when I needed them,” and “friendly, comfortable, personalized” attention. They praised the attention and engagement of the Coaches, “he was really into it” (PQ4), and mentioned how the Coaches made it easy to talk about diabetes, and could help them identify what issues they needed to discuss with a healthcare professional. Participants also spoke about their Coach remaining sensitive to their situations. For instance, participants noted that Coaches were able to adjust recommendations in the face of obstacles such as in the case of a toe complication that made it difficult to walk.

Participants noted the importance of follow-up, “I never really have anybody follow up on me. I was always disappointed with my diabetes doctor because he was very lackadaisical” (PQ5) and expressed disappointment that their healthcare providers did not review their data contained in DMA Smart Visit Reports, thereby underscoring the need to encourage clinicians to utilize DMA generated data.

As also noted in **Table 1**, participants mentioned specific behavioral changes made through being in the program (PQ6, CQ1). These fell into three categories: improving general health behaviors (e.g., quitting smoking), monitoring data (e.g., blood glucose values), and medication adherence. When explaining these behavioral changes, participants indicated that the DMA and/or the Coach helped them follow their self-management regimen.

### Complementarity

Overall, participants tended to use both DMA and Coaches as indicated by the correlation, *r* = .613 (*p* < 0.001) between DMA entries and coach contacts. However, dividing the 43 participants into those above/below the median for DMA entries and coach contacts identified several different patterns. Fourteen favored one or the other but not both of coaches or DMA (8 high coach/low DMA, 6 high DMA/low coach). Complementarity, tending to use *or not use* both if using *or not using* either, was indicated by 16 being high on both (including one missing both pre- and post-A1c values and therefore not included in **Table 4**) and, also, by 13 low on both.

All 38 participants enrolled in DMA had at least some coach contacts because of the introduction of DMA through the Coaches. Among three participants with two or fewer contacts, total DMA entries averaged 6, far below the average across all 38 DMA users of 144.14. Among four who enrolled in DMA but never entered data into it, total coach contacts averaged 3.75 with none reaching the average of 5.86 among the other 34 DMA enrollees. Thus, those with very low engagement in one of Coach or DMA tended to be low in use of the other.

Interviews also indicated complementarity of coaching and DMA: DMA “kinda guides you and the coach is a good motivator” (PQ7), “I didn’t use [DMA] for general wellness, but I used my health coach for that” (PQ8), “it’s okay [DMA] because there you put numbers and whatever you are day-to-day. The coaching is different … It’s someone that you’re listening to” (PQ9), DMA “would be more of the day-to-day, and the role of the coaching would be … discuss things that maybe aren’t so day-to-day” (CQ2). However, as 14 participants favored one of coach or DMA over the other, participants’ comments also reflected preference for one or the other as with “The only negative thing was the computer access … I think that was a little cumbersome … I just couldn’t wrap my head around it … The phone calls and the support, the coaching, and that stuff was very good” (PQ10).

DMA data reports allowed Coaches to identify participants’ self-management challenges and provided a basis to initiate conversations about those issues. Coaches noted that they tailored recommendations to participants based on data from the DMA and their conversations. Participants mentioned how Coaches helped them make sense of trends in their blood glucose and in their medication adherence, as displayed in the DMA. Furthermore, participants reported sharing reports with their Coach and discussing recipes suggested by DMA.

These quantitative and qualitative findings support the complementarity of the DMA and coaching in that use of one tended to vary in parallel with use of the other, that features of one tended to “go well with” features of the other, that they tended to go together. An alternative explanation is that individual differences such as in motivation may lead some people to do more or less of both coach contacts and DMA entries, accounting for their correlation.

### Sustainability

A major challenge for sustainability identified was the importance of the nurse coordinator or other staff to coordinate Coaches and serve as liaison to the clinical team (SQ1,2). Clearly, coaching and DMA are not self-implementing. However, one project staff member noted potential efficiency with the DMA that “has a built-in model that is sustainable.” She noted that, if Coaches could serve, e.g., 30 participants without but 50 with the DMA, “I think that’s a great argument for being more sustainable than a traditional coaching program” (SQ3). Staff also noted that efficiency would be enhanced and the burden on the care coordinator would be reduced if Coaches had direct access to EHR data (SQ4). Additional observations regarding sustainability included (a) potential to enhance integrated, value-based care with less siloing; (b) need for protocols to hire, support, and integrate Coaches into primary care practice; (c) hiring Coaches with more clinical or technical knowledge; and (d) a Coach’s observation that patient motivation remained a challenge, “trying to move people who were … not really thinking about changing from that stage to thinking about changing” (CQ3).

## Discussion

Integration of telephone-based lay health coaching with a DMA in a primary care setting was able to be implemented, accepted by individuals with T2DM, and, in uncontrolled analyses, associated with reductions in A1c. Forty-seven (50%) of 94 eligible individuals agreed to participate in the study, of whom 43 participated at least minimally. Utilization of both live coaching and DMA was frequent and positively associated, suggesting the complementarity of live and automated support—”soft touch” and “high tech.” With all participants initially selected on the basis of elevated A1c and using National Committee for Quality Assurance (42) criteria, the percentage of participants with A1c below 9% increased from 27.9 to 55.8% and below 8% from 0 to 33.3%. Additionally, those electing to participate were almost equally divided by gender (48.8% female).

In post-intervention interviews, participants expressed that they accepted the interface between participant, coach, and DMA. They expected Coaches to have access to their DMA data and use those data as part of the coaching, viewing this as a strength of the intervention. No participant mentioned feeling surveilled or disliking the monitoring by DMA. Instead, several reported liking the accountability from entering blood glucose levels in the DMA and Coaches having access to the data. As one participant put it, “… it was like someone was watching over me or I was answerable to someone. That helped me in putting in the numbers.” These comments mirror those of users of an automated telephone support intervention for diabetes management developed by Oldenburg and his colleagues (43) in Australia: “It is good to know ‘someone’ is keeping an eye (on my management).” (44)

In order to facilitate speedy training and initiation of the intervention, this study recruited lay health coaches that had prior education in promoting healthy lifestyles, behavior change, and managing chronic diseases. However, research on peer support indicates that even nonprofessionals with simple training can be effective in supporting diabetes management (5, 45). It is important to note that clinical staff perceived the Coaches having completed bachelors’ training and being enrolled in graduate nutrition programs as a strength of the intervention, linked to Coaches’ ability to be quickly deployed and capable in working with clinical staff. Though there are operational considerations that favor the recruitment of Coaches that are younger, have higher levels of formal education, and perceived to be more facile with digital health technologies, future research should explore the feasibility of using different lay health workers across the continuum of peer support (46, 47), from part-time volunteers to state-credentialed Community Health Workers.

The task of incorporating health coaches into routine clinical care may pose challenges to healthcare organizations that do not follow the patient-centered medical home model. In this study, an experienced nurse care coordinator served as bridge and coordinator among participants, Coaches, and the clinical team. This role could be standardized so that nurse care coordinators or other care managers might have the capacity to coordinate several Coaches (48–50).

The complementarity of peer support and digital health reflects a 2017 systematic review of “technology-enabled diabetes self-management” that found that the most effective interventions (a) connected people with diabetes with their healthcare teams using two-way communication, (b) analyzed participant-generated health data, (c) tailored education, and (d) individualized feedback (18). These features share much with five key functions of peer support, outlined in **Table 5**, that Peers for Progress has noted as a template for standardization and dissemination (45, 51, 52). Indeed, **Table 5** documents how digital health and live peer support complement the contributions of each to these five key functions. The key functions then provide a template for self-management support through interpersonal or technological modalities or combinations of the two.

**Table 5:** Features of peer support and Diabetes Management App (DMA) corresponding to each of the five key functions of peer support, aspects of complementarity of peer support and DMA, and quotations from participants illustrating these features.

Key Functions	Peer Support	DMA	Complementarity	Quotation
Being There	• Intrinsic in relationship • Accentuated by availability, communication skills, empathy, etc.	• Constant availability • Accentuated by sensitivity of algorithms and resources to nuanced needs of individuals	• Peer support can enhance tuning of DMA to individual needs and preferences • DMA can enhance constant and easy availability of support	**Peer Support:** “Just being there to listen to me when I was having, when it was a bad time. Just kind of talking me through maybe a different way that I can handle it.” “Persistent, not pushy” “Someone who was there for me when I needed them” “Friendly, comfortable, personalized” ***DMA:*** “Because I have to record it, it was like someone was watching over me or I was answerable to someone. That helped me in putting in the numbers.”
Assistance in Daily Management	• Personalized diabetes self-management education and support • Detailed problem-solving and long-term goal-setting • Model of adequate management	Tools for monitoring, reminders, and tracking progress • Tailored feedback effectively promote healthy behaviors • Resources for healthy lifestyles	• Key messages reinforced by coaches and DMA • Behavioral tips for healthy recipes in DMA provide discussion topics for coaching • DMA handles routine tasks of self-management so coaches can focus on complex issues	***Peer Support***: “The communication, the help, and just talking through what you have to do to try to get everything in line. Stay with your diet and exercise and take your numbers and try to keep an even keel. That’s the main thing; just try to get your basic life in order. A little more exercise, your eating habits, time of days you eat, things of that sort.” ***DMA***: “The forced regimen of checking my stuff every day twice a day. Taking the meds, that was another forced thing because I had to input it in the system when I was sure I'd taken the medication, right. That's what I was doing”
Social and Emotional Support	• Personal, supportive relationship Readily available, “being there” • Healthy coping, stress management	• “Has my back”, feeling of protection and comfort • Messages of encouragement and reassurance	• Coaches and DMA provide different sources of support • DMA alerts coaches to situations that need follow-up	***Peer Support***: “It's a job, but they still take their time to talk to you. He doesn't rush. The coach doesn't rush through the conversation. He takes his time to talk to you. It is motivational and is encouraging” ***DMA:*** “The BlueStar provided support. Especially with the little ticklers that they sent, like “oh, congratulations! Your glucose is right on target. Keep up the good work”
Linkage to Clinical Care and Community Resources	• Encouragement and reminders for clinical care • Prepare patients for clinical visits and follow up after visits • Overcome logistic, socio-economic barriers to care • Live reminders and attention to psychosocial barriers to care	• SMART Visit Reports give insights into participants’ self-management needs • Monitoring provides automated, specific reminders for routine care or care as needed • Geocoded availability of restaurants, other resources	• Coaches and DMA work together to reinforce importance of primary care and specialty referrals • DMA SMART Visit Reports help guide discussions with coaches and providers	***Peer Support***: “[My health coach] was great. She's a good listener. Also, too she's very encouraging. If you hit a little spot where you're talking about stubborn blood-sugar readings and stuff like that, she'll encourage you to maybe talk to your doctor, your nutritionist, and that kinda stuff. Just not give up.”
Ongoing Support	• Available on demand • Quarterly “check-in”; more frequent messaging	• Availability 24/7 • Available indefinitely with down or up titration as needed • Continued reimbursement contingent on continued use	• Coaches encourage continued use of DMA	***Peer Support:*** “Basically just to try to help me bring down the targets, where they should be, and pretty much was my intake. He said reduce it. He kinda watched me on a personal level, from my eating habits, to pushing me on the exercising, and just kind of ... It's almost like, you know what you gotta do, and you gotta be pushed to do it, but you gotta get motivated. I think he did a good job of trying to motivate me over the entire study period. He was always there. Sometimes it's not easy to do, because there's fifty million other things you do in the day. You know?” ***DMA***: “The BlueStar, when you take a blood-sugar reading, you can just enter it the information in, you can make notes, there are a lot of things you can do. It's very good that way. Log books sometimes you don't have space to do anything. You've gotta have a pen, you've gotta have a book. I just like having a single item that I can use to do all of this… I've been using it every, basically every day. I'd have to give it a 10.”

The present observations that participants saw the DMA and live coaching as fitting well together suggest that digital health apps may be considered a form of automated, resource-saving, low-cost support, ready to be paired with live peer support for those with greater need, e.g., suboptimal self-management, complex multi-morbidities, or psychosocial concerns. Extending such thinking beyond the details of this study, population management might titrate app use and live coaching (53). As an example of this kind or approach, a simple model might include *Level One: Doing well* (e.g., A1c < 7%, no psychosocial concerns)—Routine availability of app; *Level Two: Little clinical or psychosocial concern* (e.g., A1c < 7.5%, no pronounced psychosocial concerns)—Routine encouragement of app through primary care visits; *Level Three: Moderate clinical concern* (e.g., A1c > 7.5%)—Limited coach contacts to support self-management and encourage use of app; and *Level Four: Substantial clinical concern* (e.g., A1c > 8% and/or appreciable psychosocial distress)—Live coaching for 6 months, renewable as needed, with incorporation of app as acceptable or helpful. Although they surely do not provide adequate data for concluding that such a model would be effective, the present study results suggest that this type of titration of interventions may be realistic and worthy of future research.

Digital health is “what’s coming” across a range of functions and services. With mobile health apps available not only for wellness support but also for most common conditions, there are solutions available to support each step of the journey of those with diabetes on devices that people already own. Experts have noted the remarkable market-driven growth of mobile health apps for diabetes (54, 55). People with diabetes, clinicians, investors, and tech developers all seem to recognize the promise of digital health in diabetes care, and regulators are encouraging growth in this field by lowering barriers to accessing new technologies (56).

The issue is not *if* but *how* to deliver digital health to individuals and integrate it with clinical care. Health coaches may be a valuable strategy in rolling out digital health to patient populations. From the perspective of digital health, an important contribution of health coaching is how it promoted the uptake and *continued use* of DMA. From the perspective of the health coaches, DMA provided a focal point for participant monitoring and generating discussion, which helped to shape participant encounters and pinpoint key areas of improvement for each individual participant. In addition to more rigorous, controlled tests of efficacy and effectiveness, follow-up studies might examine how digital health solutions impact the ways in which Coaches deliver diabetes support, such as by comparing how Coaches adjust their counseling strategies when assisted by digital health solutions versus when using traditional strategies. In addition, future developments might involve adding in-person coaching, in either individual or group formats.

### Limitations

In this study, participants were encouraged to engage with service offerings as they wished, similar to the way in which comprehensive chronic disease programs might be implemented under real-world conditions. One limitation of the study is the selection bias for participants who are interested in digital health and have the means and skills to use such tools.

The intervention was planned to meet the needs of those having trouble managing their diabetes (mean baseline A1c = 9.93%) and the sample had, on average, incomes estimated at 31.1% below the statewide average. However, the intervention was not tailored to any specific racial or ethnic group, perhaps limiting generality to some groups with disproportionate diabetes burden. At the time of this study, the DMA was only available in English, which excluded individuals not fluent in English.

Other limitations of the project include the relatively small sample size and the lack of controls. Additionally, participant data were drawn from EMR records as part of routine care instead of being gathered as part of an independent collection of evaluation data.

### Conclusions

Contributing to the growing research around digital health for diabetes management, this study shows that integrated health coaching/DMA is feasible and acceptable for individuals with diabetes, coaches, and clinicians. The findings suggest that these two approaches complement each other, meriting additional studies to test integrated models at scale and address implementation challenges for clinical and organizational adoption.

## Data Availability Statement

The datasets presented in this article are not readily available because of the modest size of the sample, and because the data consist largely of clinical data, we would anticipate problems in assuring confidentiality of participants’ data and, so, have decided not to make the data available. Questions about details of the data may be directed to PT (ptang@unc.edu) or EF (edfisher@unc.edu). Requests to access the datasets should be directed to PT (ptang@unc.edu) and EF (edfisher@unc.edu).

## Ethics Statement

The studies involving human participants were reviewed and approved by the Institutional Review Board at the University of North Carolina at Chapel Hill. The patients/participants provided their written informed consent to participate in this study.

## Author Contributions

PT, JD, MP, and EF led the development, implementation, and evaluation of the project and the preparation of the manuscript. SK, NB, and RS contributed to planning the implementation and evaluation of the project, and contributed to conducting evaluation including data management, data analysis, and coding and interpretation of interviews. All authors contributed to the article and approved the submitted version.

## Funding

Funding for this project was provided by a Gillings Innovation Laboratory award funded by the 2007 Gillings Gift to UNC-Chapel Hill’s Gillings School of Global Public Health. The funding source had no role in the study design; data collection; administration of the interventions; analysis, interpretation, reporting of data; or decision to submit the findings for publication.

## Conflict of Interest

Subsequent to completion of this study, EF and PT became part of a contract with WellDoc that provided BlueStar^®^ Diabetes for the present study. The contract was for development of programs in areas other than diabetes. MP is an employee of WellDoc that provided the BlueStar^®^ Diabetes for the study. She collaborated throughout planning, implementation, and preparation of this manuscript, and specifically contributed to the description of BlueStar^®^ Diabetes and product user data.

The remaining authors declare that the research was conducted in the absence of any commercial or financial relationships that could be construed as a potential conflict of interest.

## Publisher’s Note

All claims expressed in this article are solely those of the authors and do not necessarily represent those of their affiliated organizations, or those of the publisher, the editors and the reviewers. Any product that may be evaluated in this article, or claim that may be made by its manufacturer, is not guaranteed or endorsed by the publisher.
